# Activation of Cellular Players in Adaptive Immunity via Exogenous Delivery of Tumor Cell Lysates

**DOI:** 10.3390/pharmaceutics14071358

**Published:** 2022-06-27

**Authors:** Jihyun Seong, Kyobum Kim

**Affiliations:** Department of Chemical and Biochemical Engineering, Dongguk University, 30 Pildong-ro 1-gil, Jung-gu, Seoul 22012, Korea; jh.seong520@gmail.com

**Keywords:** tumor cell lysate, adjuvant, dendritic cell, exogenous delivery system, cancer immunotherapy

## Abstract

Tumor cell lysates (TCLs) are a good immunogenic source of tumor-associated antigens. Since whole necrotic TCLs can enhance the maturation and antigen-presenting ability of dendritic cells (DCs), multiple strategies for the exogenous delivery of TCLs have been investigated as novel cancer immunotherapeutic solutions. The TCL-mediated induction of DC maturation and the subsequent immunological response could be improved by utilizing various material-based carriers. Enhanced antitumor immunity and cancer vaccination efficacy could be eventually achieved through the in vivo administration of TCLs. Therefore, (1) important engineering methodologies to prepare antigen-containing TCLs, (2) current therapeutic approaches using TCL-mediated DC activation, and (3) the significant sequential mechanism of DC-based signaling and stimulation in adaptive immunity are summarized in this review. More importantly, the recently reported developments in biomaterial-based exogenous TCL delivery platforms and co-delivery strategies with adjuvants for effective cancer vaccination and antitumor effects are emphasized.

## 1. Introduction

Cancer immunotherapy is an emerging antitumor treatment technique, which works via specific antigen-mediated modulation in the patient’s immune system [1]. Conventional anticancer therapies, including chemotherapeutic drugs and targeted treatments, have clinical limitations and adverse effects, such as non-specificity, drug resistance, and low efficacy in cancer mutation and metastasis [2,3,4]. Hence, the precise control and modulation of adaptive immune responses for pre-existing intratumoral therapy is the most important engineering parameter for developing effective cancer immunotherapeutic approaches [5,6,7].

**Figure 1:**
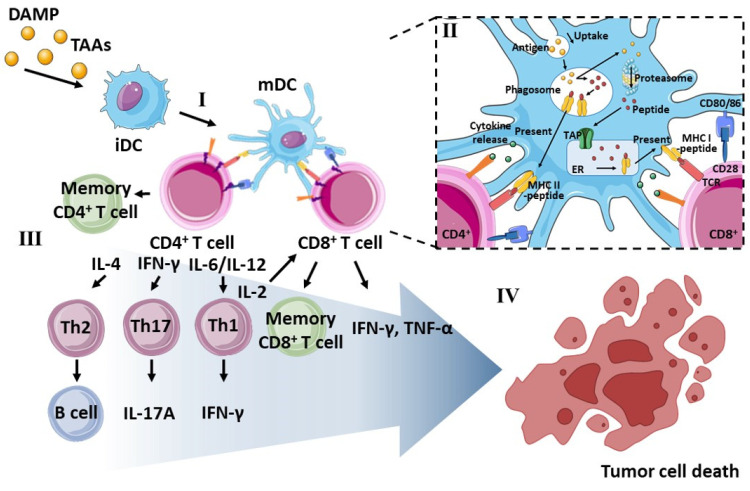
Overview of DC and T cell interplay for anticancer immunotherapy. (**I**) Differentiation of stimulated immature DCs (iDCs) by DAMP molecules and TAAs to mature DCs (mDCs), (**Ⅱ**) process of antigen presentation via MHC molecules in DCs, (**Ⅲ**) priming of T cells to effector T cells, (**Ⅳ**) induction of the tumor cell death by various types of T cells. Reproduced with permission from [10]; images used are from Servier Medical Art.

Based on the interplay between T cell populations and other immune cellular components, engineering modulation in adaptive immunity could effectively eliminate cancer cells and inhibit tumor growth. As depicted in Figure 1, in particular, CD4^+^ T cells differentiate into various T helper cell subsets, including T helper (Th)1, Th2, Th9, Th17, and T follicular helper cells, in which Th1 cells react with antigen-presenting cells (APCs) and indirectly assist in the differentiation of CD8^+^ T cells into cytotoxic T lymphocytes (CTLs) by secreting a cytokine, such as interferon (INF)-γ. Additionally, interleukin (IL)-2 secreted from Th1 induces the proliferation of CD8^+^ T cells [8,9]. Thus, both activated CD4^+^ and CD8^+^ T cells augment long-lasting and strong antitumor immune responses by generating memory T cells to persist in anamnestic immune responses. APCs such as macrophages and dendritic cells (DCs) are crucial mediators for inducing T cell activation by antigen presentation on their surfaces. After taking up the tumor antigen molecules, these antigens are processed in the proteasome or phagosomes in the cytosol and presented in the form of peptides via major histocompatibility complex (MHC) class I or II molecules on the cellular surface of APCs. APCs with the MHC–peptide complex travel to secondary lymphoid organs to stimulate T cells. Upon contacting T cells, APCs initiate the priming of naïve T cells by interacting with the MHC–peptide complex and T cell receptor (TCR), and secrete cytokines to activate T cells.

Previous studies have experimentally demonstrated that APC–T cell interaction by three distinct signals effectively induced antigen-specific T cell activation [11,12]: (1) the interaction between antigenic peptides presented by the MHC and the TCR; (2) co-stimulatory signals induced by the interaction between B7 molecules (e.g., CD80 and CD86) in APCs and CD28 in T cells, which trigger stronger immune responses; and (3) polarizing signals mediated by the production and secretion of multiple cytokines (e.g., IL-12 and tumor necrosis factor (TNF)-α by APCs [13]. Among these signaling interactions, MHC-mediated antigen cross-presentation is the most critical for the initiation of antigen-specific immune responses. Presented MHC I-immunogenic peptide complexes can be recognized by CTLs [14], whereas T helper cells can be activated by MHC II-mediated extracellular (or exogenous), immunogenic, antigenic peptide complex presentation [15]. Finally, activated T cell populations migrate toward the tumor microenvironment to kill specific tumor cells [16]. Cancer cells overexpress universal tumor-associated antigens (TAAs) and individual mutant neo-antigens [17,18]. To incorporate these TAAs for facilitating APC-dependent antigen presentation and subsequent T cell activation, lysed tumor cell bodies containing soluble tumor antigen molecules (e.g., tumor cell lysates (TCLs)) have been investigated for cancer therapy.

Therefore, cell-based engineering techniques to control and stimulate adaptive immune responses and further tumor suppression have recently been developed for designing efficient cancer therapeutics and vaccinations. TCLs containing various epitope sources are utilized for the induction of both CD8^+^ and CD4^+^ T cells [19,20] and potential personalized therapy. Endogenous damage-associated molecular patterns (DAMPs) are released by dying or damaged cells (i.e., host biomolecules that can initiate non-inflammatory responses to infection), and these specific TAAs interact with the pattern recognition receptors (PRRs) of DCs. Sequentially, activated antigen presentation on DCs induces proper T cell priming toward Th1 cells and differentiation of T lymphocytes into CTLs. Moreover, secreted cytokines, such as IL-12, IL-15, and IFN, from DCs are also able to stimulate T cell activation [20]. These T cells primed by APCs with TCL-derived antigens are key effectors of anticancer immunity. Antigen-specific memory T cells, which exert immediate effector functions without the need for further differentiation, sufficiently suppress tumor recurrence [21].

A growing body of research has explored the potential capability of TCLs for cancer vaccination. Because of the high antitumor immunity effects of TCLs, the majority of recent cancer immunotherapies utilize the TCL-mediated activation of APCs and T cells, along with the MHC pathway. However, due to the technical drawbacks of the naked form of TCLs, including a short half-life and the limited availability of various antigens, a lower therapeutic effect in immunity than the treatment of a specific antigen has been frequently observed. To strengthen the clinical efficacy of TCLs, particularly in the case of in vivo administration, precisely designed delivery systems should be utilized for increasing the stability of cargo TCLs and facilitating the co-administration of adjuvants. Therefore, the TAA-mediated activation of TCLs as an immune activator could be applied to induce in vitro necrosis of cancer cells, and stimulate downstream antitumor responses and immunological memory generation.

To this end, this review focuses on the current progress in engineered cancer immunotherapies by exogenous TCL delivery, emphasizing (1) practical applications using TCL-mediated DC activation and sequential stimulation in adaptive immunity in various cancer types, (2) the significance of adaptive immunological functions, and (3) the utilization of a series of delivery platforms for the co-administration of multiple adjuvants for effective cancer vaccination and antitumor treatment.

## 2. Preparation of Tumor Cell Lysates

### 2.1. Physical Disruption and Stimulation of Tumor Cells to Obtain Whole Tumor Cells

**Figure 2:**
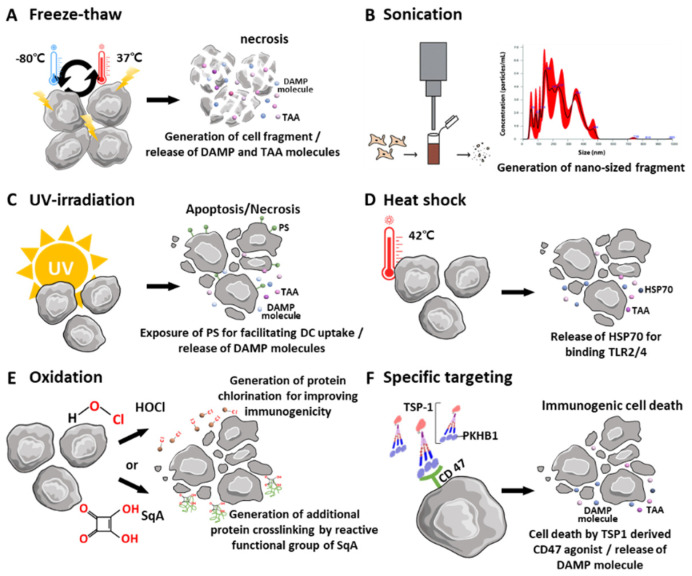
Schematic illustration of preparation of TCLs via various conditions. (**A**) Repeated freeze–thaw cycle could induce necrosis of tumor cells and release immunogenetic molecules (e.g., DAMPs, TAAs), (**B**) sonication generates nano-sized fragments, average particle size as measured by Nano Sight, (**C**) UV irradiation for facilitated cellular uptake, (**D**) heat shock evokes release of HSP70 in tumor cells, (**E**) oxidation rapidly induces tumor cell death and generates advantageous immunogenic molecules, (**F**) PKHB1 peptide-derived TSP-1 interacts with CD47 and activates the atypical caspase-independent and calcium-dependent signaling in cell death. (**B**,**F**) are reproduced with permission from Refs. [29,33,36].

TCLs prime antitumor immunity and exhibit immune tolerance against self-antigens. Live tumor cells as a source of antigens could be less immunogenic since these cells contain or secrete factors such as vascular endothelial growth factor, soluble FAS ligand, and MHC class I chain-related proteins A and B, which suppress the function of DCs and T cells [7]. Figure 2 demonstrates the summary of cell lysis by external factors, and their conditions are indicated in Table 1.

Simply, TCLs are generated by repeated freeze-and-thaw cycles (Figure 2A), and protein fragments from the whole tumor cell population are obtained. The development of ice crystals during freezing, and the subsequent concentration upon thawing, results in the physical rupture of cellular bodies [37]. This repeated process facilitates the large-scale release of inflammatory proteins [38]. In general, these protein fragments are DAMPs, including heat shock protein (HSP) and high-mobility group box-1 (HMGB-1), which are classified as class I DAMPs [39]. HSPs and HMGB-1 directly bind and trigger Toll-like receptor (TLR) 2 and TLR4, which are the PRRs located in immune cell membranes. Activated TLR2 and 4 initiate NF-kB and interferon regulatory factors via the myeloid differentiation primary response 88-dependent pathway and toll/interleukin-1 receptor domain-containing adapter-inducing interferon-ß-dependent pathways. Through this, pro-inflammatory cytokines (e.g., IL-1ß, IL-6, and IFN) are released from DCs and stimulate T cell immunity with presented tumor-specific antigens using MHC molecules. [40].

The subsequent physical treatment of tumor cells, such as sonication, is optionally introduced to facilitate the homogeneity of the prepared TCLs. Nano-scale TCLs could be obtained using only sonication (Figure 2B) [29]. Additionally, ultraviolet (UV) irradiation is also commonly used to prepare TCLs by inducing immunogenic cell death (ICD) (Figure 2C) [30]. UV irradiation (1500 μW/cm^2^ for 10 min) of TC-1 tumor cells results in both apoptosis and necrosis, and the TCLs from these UV-pulsed DCs exhibit significant surface expression of CD86, CD80, and MHC II molecules. The UV irradiation of tumor cells also generates effective TCL modulators for inducing an antitumor immune response by further enhancing CD8^+^ cell populations.

### 2.2. Pretreatment of Source Tumor Cells

#### 2.2.1. Heat Shock

The induction of early necrosis using heat shock could be an alternative approach to obtaining TCLs (Figure 2D). A temperature of 42–43 °C could induce optimal cell death in antitumor immune outcomes, and maximum HSP production in the extracellular spaces of necrotic tumor cells, which could activate an adaptive antitumor immune response [41]. Mild hyperthermia (around 40 °C) induces thermotolerance [42], whereas high hyperthermia (over 45 °C) induces protein denaturation. In particular, HSP70 directly binds to CD40 receptors of DCs, and promotes the release of co-stimulatory signals [43]. Heat treatment of tumor cells also increases the expression of other DAMPs (such as HMGB-1 and ATP), and these molecules are recognized as danger signals by DCs.

For example, the heat shock treatment of three human melanoma cell lines at 42 °C for 1 h resulted in an allogeneic TCL mixture (TRIMEL) containing antigen components. The administration of TRIMEL significantly upregulated the release of the pro-inflammatory cytokine IFN-γ in DCs compared to the application of TCLs without heat shock treatment. Consequently, a previous study reported that TRIMEL showed clinical vaccination effects by developing a delayed type of hypersensitivity response in 64% of patients [31].

#### 2.2.2. Oxidation

The oxidation of source tumor cells prior to the preparation of TCLs could facilitate necrosis and augment the immunogenicity of the antigenic components in TCLs by increasing oxidative stress (Figure 2E). Through this modulation, DCs could boost the uptake of antigenic danger signals and antigen processing mechanisms [44]. Therefore, hypochlorous acid (HOCl)-mediated oxidation is used for the generation of effective TCL contents, since protein chlorination enhances proteolytic vulnerability and improves the immunogenicity of the antigenic components [45]. HOCl-mediated oxidation also produces aldehyde-modified antigens with higher immunogenicity than that of unmodified antigens [46]. Chiang et al. [47] compared the in vitro efficacy of DCs pulsed with various TCLs obtained by HOCl-mediated oxidation, UVB irradiation, and six freeze–thaw cycles. Here, both HOCl-mediated oxidation and UVB irradiation efficiently induced the necrosis of tumor cells expressing ovalbumin (OVA) antigens, and the MHC-1-dependent presentation of the peptide SIINFEKL was achieved in DCs treated with the TCLs. In vivo tumor suppression in ID8 ovarian tumor models also demonstrated the enhanced immunogenic capability of the antigen contents in TCLs obtained from HOCl-mediated oxidation.

As a more stable molecule than HOCl, squaric acid (SqA) has been clinically approved for the treatment of skin papillomas [32]. SqA was also shown to induce the complete necrosis of source tumor cells and induce subsequent chemical changes in tumor antigens by combining with them via mechanisms including redox alteration, additional crosslinking, and aggregation through the reactive functional group. The resulting DAMPs from SqA-treated TCLs stimulated DCs, and these activated DCs elicited significant cytokine (IL-12 and IFN-γ) secretion and antigen presentation ability, indicating a more potent Th1 response.

#### 2.2.3. Specific Targeting

Furthermore, the incorporation of biological substances into source tumor cells could also augment TCL-mediated immune activation. One of these stimulatory substances is known to act as an agonist peptide to activate CD47 in cancer cells. Previous reports demonstrated that CD47 activation using soluble peptides derived from thrombospondin-1(TSP-1) effectively induced cell death in several types of cancer cells (Figure 2F) [48,49]. Particularly, ICD induced by a TSP1-derived CD47 agonist (PKHB1 peptide: KRFYVVMWKK), and DC activation using TCLs obtained from PKHB1-treated L5178YR tumor cells (PKHB1-TCL), has been reported [33]. The sequential mechanisms of (1) CD47 activation by PKHB1, (2) exposure to several DAMPs by atypical caspase-independent and calcium-dependent signaling in cell death, (3) the enhanced maturation of bone marrow-derived DCs with proper antigen presentation, and (4) the stimulation of antitumor T cell responses in an in vivo L5178Y-R tumor model using syngeneic BALB/c mice, were obtained using PKHB1-TCLs.

#### 2.2.4. Treatment with Natural Compounds

A natural compound was also used to modulate source tumor cells to facilitate the apoptosis of cancer cells. Pretreatment of both HCT 116 and MCF-7 cancer cell lines with an ethanol extract of *Phyllanthus amarus* induced the reactive oxygen species (ROS)-mediated apoptosis of tumor cells [34]. The TCLs from these apoptotic cancer cells effectively activated monocyte-derived DCs, showing significantly facilitated gene expression levels of IL-12 and IL-6 cytokines compared to TCLs from lipopolysaccharide (LPS)-treated cancer cells. The subsequent maturation of DCs was also determined by the enhanced immune functions of antigen presentation, chemotaxis capacity, phagocytic activity, T cell proliferation, and cytokine release.

### 2.3. Preparation of Tumor Cell Membranes

**Figure 3:**
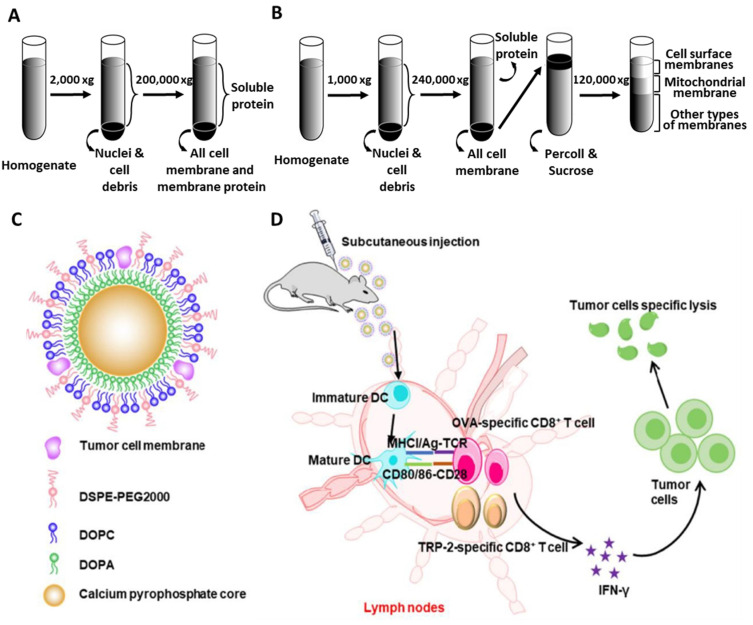
Schematic illustration of tumor cell membrane isolation and application in anticancer immunotherapy. (**A**) Cell membrane isolation using ultracentrifugation to obtain the total cell membrane component, (**B**) sucrose-dependent isolation for separating the cell surface membrane components, (**C**) design of tumor cell membrane-coated CaPyro nanoparticle, and (**D**) anticancer immunity mechanism using these NPs. All subfigures were reproduced with permission from Refs. [10,51].

As a source of TCLs, several studies primarily focused on the production of tumor cell membrane components. Since cell membrane contents participate in protein–protein interactions in immune system processes, inflammatory responses, and chemokine signaling pathways, tumor cell membrane proteins (e.g., CD44, MUC, CD98, and integrin) could be used as tumor-specific antigens and receptors to effectively trigger immune responses in cancer therapy [50]. Centrifugation has been used to isolate cell membrane proteins from tumor cells. For purification, (1) the physical disruption of collected cells by homogenization or freeze–thaw cycles with lysis buffer, (2) centrifugation at low speed (1000–2000 RCF) to separate cellular debris and nuclei contained in the pellet, and (3) ultracentrifugation at high speed (100,000–200,000 RCF) to separate all membrane fractions from soluble proteins in the supernatant, have generally been performed (Figure 3A). Additional sucrose treatment provides a density gradient for obtaining membrane fractions (Figure 3B). The resuspension of membrane fractions within sucrose results in further separation of cell surface membranes, mitochondrial membranes, and other types of membrane components [51]. Isolated cell membrane components obtained through this centrifugation process could be further incorporated into various template biomaterials. For instance, a biomimetic antitumor nanovaccine was fabricated via the coating of membrane components onto calcium pyrophosphate inorganic NP templates (Figure 3C) [10]. This inorganic carrier platform to deliver TCL membrane-derived antigens consisted of (1) cell membrane fragments, isolated from sucrose-dependent separation, that promoted specific immune reactions as antigens, and (2) biocompatible calcium phosphate templates as immune adjuvants that stimulated innate immunity by activating the NLRP-3 inflammasome and the production of cytokines (e.g., IL-1ß) [52] for T cell-based responses. Therefore, the dual functionality of calcium pyrophosphate nanoparticles coated with antigen-rich TCL membranes could improve the antigen presentation of DCs, as well as provide adjuvant effects, dramatically increasing the expression of DC surface markers and the subsequent proliferation of CD8^+^ T cells (Figure 3D).

## 3. Role of DCs in Cancer Immunotherapy

### 3.1. Phenotype of Dendritic Cells

DCs located in the spleen and various lymphoid tissues generally exhibit unique immune functions in activating T cells through antigen presentation [53,54]. However, the interaction between DCs and T cells occurs only in mature stages of DCs, which depends upon successful antigen uptake. DCs mostly exist in an immature state, but sufficient antigen uptake initiates a change to the mature state. During the functional maturation process, changes in the morphological and phenotypic characteristics of DCs influence immune system activity [55]. Mature DCs (mDCs) with a rough surface and multiple pseudopodia, and immature DCs (iDCs) with a spherical and smooth structure, exhibit different phagocytotic and migration abilities [56]. Therefore, when phagocytosis and endocytosis preferentially occur in the immature state, the morphological conversion (i.e., more dendritic structure) and optimization of antigen presentation by DCs occur sequentially. Then, these mDCs with a higher level of MHC molecules quickly migrate to the lymph nodes for 2–3 days, while maintaining their presentation ability, and are ready to stimulate other immune cells [57]. Consequently, mDCs can initiate and maintain adaptive immunity (including antigen specificity, humoral immunity mediated by antibodies, antigen-specific cellular immunity and memory) through a pathophysiological network with other immune cells, such as T cells, B cells, and NK cells [58].

### 3.2. Antigen Presentation by MHC Molecules

The recognition of MHC I and II molecules is crucial for the communication that leads to DC-induced immune responses. The major MHC-dependent antigen process in DCs can be identified as followed: MHC II aids in the presentation of exogenous antigens internalized into DCs, whereas MHC I helps in the presentation of peptides generated from reprocessed proteins and peptides through proteasome-mediated degradation in the cytosol [59,60,61].

DCs provide pathogenic information that “alerts” the immune system to an infection by increasing MHC II production, or regulating MHC II degradation, by the following mechanisms [62]: (1) after synthesis in the endoplasmic reticulum (ER) of APCs, MHC II molecules are delivered to the plasma through the Golgi network, or by direct transport to late endosomal compartments, (2) plasma-loaded MHC II molecules internalize exogenous protein antigens by clathrin-mediated endocytosis [63], (3) the internalized antigenic proteins are processed to peptides via endosomal and lysosomal proteolysis, (4) the processed peptide molecules are then combined with MHC II on the late endosomal surface, and these immunodominant MHC II–peptide complexes migrate to the cellular surface membranes of APCs for identification by CD4^+^ T lymphocytes, and the initiation of T helper immune responses [64,65], and (5) MHC II–peptide complexes are recycled through ubiquitination in proteasomes, and further degradation processes in lysosomes, until DC maturation is complete [66,67].

MHC I-mediated cross-presentation in the immune system occurs via immune proteasomes [68]. For the cross-presentation of exogenous TAAs using MHC I molecules: (1) exogenous antigenic proteins (such as viral proteins produced during infection) internalized by phagocytosis are transferred to proteasomes via the ubiquitin–proteasome pathway, and degraded by proteolytic enzymes; (2) the resulting peptides are transported into the ER by the transporter associated with antigen processing (TAP) [69] and an ATP-dependent transporter; (3) MHC I molecules are fabricated in the ER and connected with the TAP, and subsequent binding of MHC I to the transported peptides occurs; (4) MHC I–peptide complexes are then delivered to cell surface membranes for cross-presentation to activate antigen-specific CTLs; and (5) completely equipped CTLs kill prospective target cells, such as virus-infected cells or tumor cells [70]. Although it does not contribute as much as the proteasome pathway, the vacuolar pathway, which does not rely on proteasomes and TAP, also participates in cross-presentation via MHC I [71]: (1) internalized exogenous antigens are degraded by protein catabolism using cathepsin S as a protease within the endocytic compartment, (2) MHC I molecules are generated from the ER and transferred to the endosome, and (3) MHC I-containing endosomes are loaded with the peptides, and then, peptide–MHC I complexes are presented on the cellular plasma membrane [72].

### 3.3. Downstream T Cell Commitment by mDCs

After successful antigen presentation by DCs, the interaction between mDCs and T cells in lymph nodes occurs to initiate cell-mediated adaptive immune responses. Further T cell commitments, such as proliferation and differentiation, are regulated by the level of TCRs triggered by antigen-presenting mDCs and the effectiveness of the signal amplification the T cells receive [57]. Several types of important mDC-mediated signals in lymph nodes are required for the activation and differentiation of naïve T cells. (1) The peptide–MHC complex initiates antigen-dependent signal transduction, (2) costimulatory molecules (i.e., B7 molecules, CD40, or ICAM-1) amplify the signaling process, and even a low level of available antigens effectively induces TCR-dependent T cell commitment [73,74], and (3) soluble cytokines facilitate further T cell activity

One of the crucial cytokine signals, IL-2 produced by activated Th1 cells, upregulates T cell proliferation. The direct activation of CD8^+^ T cells, and the subsequent expansion of T cell populations upon TCR activation, is mediated by autocrine and paracrine IL-2 signaling [75,76]. IL-2 also promotes the differentiation of effector T cells [77]. Moreover, the duration of sustained TCR stimulation is controlled by the secretion of IL-12 by mDCs, which promote the progression of T cell differentiation and the subsequent formation of terminally differentiated effector cells. Specifically, in the presence of IL-12, T cells can develop into Th1 cells or Th2 cells, and these T helper cell populations gain the ability to move to inflamed organs to perform their own roles as effectors [78]. The stability of the mDC–T cell synapse maintains the duration of the stimulation during the signaling and transduction processes [79]. For instance, CD4^+^ T cells need to be in contact with mDCs for 24 h to induce efficient cell division [80]. Even when naïve CD8^+^ T lymphocytes interacted with mDCs for only 8 h, they exhibited a stronger proclivity for differentiating into effector and memory T cells [81,82].

### 3.4. Limitations of Ex Vivo Manipulation and the In Vivo Administration of DCs

Previous immunotherapeutic strategies have used the direct administration of ex vivo pulsed autologous DCs to activate T cell populations. One of the representative APC-based administrations was first approved by the Food and Drug Administration (FDA) as a cancer vaccine (Sipuleucel-T; Dendreon, CA, USA) in 2010 for late-stage castration refractory prostate cancer. This method includes APC isolation from patient blood, the co-incubation of APCs with prostatic acid phosphatase antigen and a granulocyte-macrophage colony-stimulating factor (GM-CSF), and reperfusion into the patient [17]. To develop an engineering manipulation of DCs using whole TCLs, the ex vivo differentiation of monocyte-derived autologous DCs was achieved by the incorporation of GM-CSF, IL-4, and additional stimuli components (e.g., LPS or TNF-α) to increase the potency of DC activation. Pulsing DCs by incubating them with TCLs also facilitates the production of mDCs [83,84]. Therefore, the vaccination platform involving ex vivo DC pulsing has also been applied to several cancer types, and the potential immunological response against specific cancers, with suitable safety for clinical trials, has been demonstrated. The ex vivo manipulation of DCs using melanoma-derived antigenic TCLs effectively induced signals for melanoma-associated antigen-1 (MAGE-1)-specific CTL responses, and two out of sixteen patients showed long-lasting immune responses over 6 months by successfully modulating antitumor immunity [85]. When using a HOCl-treated TCL mixture (derived from three ovarian tumor lines), DCs also exhibited Th1-dependent antitumor effects and tumor growth delays in stage II/IV ovarian cancer patients [47].

However, the therapeutic efficacy of the ex vivo manipulation and in vivo administration of DCs depends upon the administration route [86], sufficient numbers of delivered DCs [87], and the DC subset [88,89]. It has been reported that more than 90% of ex vivo engineered DCs died or were lost to non-targeted sites, and therefore, only a small fraction of the delivered DCs could home in on a lymph node, resulting in an insufficient T cell response [90,91]. Additionally, the optimization of ex vivo culture conditions, the expansion process, and the loading efficiency of tumor antigens for proper antigen presentation, are all required [92,93]. Due to these technical limitations in obtaining sufficient in vivo immune responses, direct injections of TCLs targeting in vivo resident DC populations without ex vivo DC control have been extensively studied to facilitate antigen-specific immune responses against cancer. Therefore, recent progress in biomaterial-mediated in vivo TCL administration and successful T cell pathway activation by antigen-presenting DCs are emphasized in the following sections.

## 4. Therapeutic Outcomes of Exogenous TCL Delivery Using Various Biomaterials

Exogenous TCL delivery using various multifunctional biomaterials through in vivo administration has been utilized in cancer immunotherapy. In particular, exogenous TCL delivery induced APC-dependent enhancement and the effective orchestration of adaptive immune responses by (1) augmenting in vivo DC maturation and activation, (2) increasing antigen presentation in DCs, and (3) further inducing T cells by interacting with multiple DCs. However, weak immunogenicity can be caused by a variety of factors, including (1) a lack of appropriate immunological DAMP signals [94], (2) inefficient delivery of relevant TAAs to resident in vivo DCs, and (3) the undesired degradation of antigen molecules during migration in the bloodstream and lymphatic system. Therefore, various delivery platforms with protective efficacy for cargo TCLs, and additional functionality to improve the immunogenicity of TCLs, have been investigated. As well as the intrinsic stimulation by biomaterial via direct immune cell regulation (i.e., DC activation and T cell proliferation) through the recognition of exogenous substances and their following interactions with immune cells [95], cargo TCL protection (i.e., preservation of its bioactivity upon in vivo administration) and subsequent augmentation under sustained DC activation, are technical advantages of biomaterial-based delivery platforms. The successful development of an efficient TCL delivery platform could represent a novel immune modulatory strategy for anticancer treatments, through the sequential accurate targeting processes of longer circulation with improved colloidal stability, sufficient delivery of TAAs in TCLs to lymph nodes, the sustained release of cargo TCLs, preservation of in vivo TCL bioactivity, and the enhanced cellular uptake of TAAs to DCs [96,97]. Hence, recent therapeutic approaches have focused on material-assisted TCL delivery platforms. This section reviews the current progress in TCL-mediated immune activation, anticancer treatment, and prospective applications of cancer vaccines.

### 4.1. Nanoparticles

#### 4.1.1. Design Parameters for TCL Carriers

The most representative TCL delivery platform comprises nanoparticle (NP)-based carriers, which use several types of materials decorated with functional moieties to boost their delivery efficacy. The encapsulation of TCLs in the NP core can protect cargos from degradation during in vivo circulation, and regulate their release. These particle-based carriers can also be easily modified with functional ligands or molecules on their surface [98,99]. The efficiency of antigen-containing TCLs in draining lymph nodes can be influenced by the characteristics of the template particles, such as size, morphology, and charge.

For example, the efficiency of nano-sized polystyrene particles for activating APC subsets was higher than that of micro-sized particles in terms of cellular uptake [100]. NPs can easily infiltrate cells, and their resident particle populations in lymph nodes are three fold larger than those of micro-sized particles. Thus, a potential T cell immune response can be effectively induced by DC maturation. Moreover, this size-dependent immunogenicity was also observed in vaccination efficacy against tumors [101]. The delivery of human papillomavirus peptides using 40–50 nm Ag NPs resulted in higher uptake into DCs in draining lymph nodes, in vivo localization in C57BL/6 mice models, and immunological responses. Protection in tumor challenge models and the clearance of established tumors was also found.

In terms of the morphology and geometry of particulate carriers, the spherical shape of NPs exhibits (1) reduced adhesion to vessel walls, and longer circulation time [102], and (2) facile ligand conjugation onto larger surface areas. Spherical NPs with surface-conjugated ligands can be fully enveloped by target cellular membranes via strong ligand–receptor interactions, and consequently facilitate receptor-mediated endocytosis [103]. Spherical NPs could overcome a minimal membrane binding energy barrier, resulting in low free energy change for internalization into target cells [104]. Shape-dependent immune adjuvant efficacy was also observed in the delivery of AuNPs coated with virus envelope proteins (VEPs) [105]. The in vivo inoculation of these NP-VEPs into mice resulted in shape-dependent cytokine production in DCs. Rod-shaped NPs induced pro-inflammatory IL-1β and IL-18 production by activating the inflammasome-dependent process as adjuvants for eliciting immunity. In contrast, the same antigen delivery system using spherical or cube-shaped NPs induced the secretion of other types of inflammatory cytokines, including TNF-R, IL-6, IL-12, and GM-CSF.

An optimal surface charge and charge density of NPs is also required in order to increase the duration of blood circulation and prevent their loss to untargeted regions [106]. In general, positively charged NPs interact more efficiently with negatively charged cell membranes, and higher cellular uptake occurs [107]. A previous study reported charge-dependent NP uptake by 3T3 fibroblasts [108], indicating that the cellular internalization of trimethylammonium-coated AuNPs with positive surface charges was faster than that of negatively charged phosphonate-coated particles. The interaction with various in vivo protein components and delivered NPs resulted in the formation of a protein corona, which might reduce NP uptake regardless of the charge of the NPs. Moreover, a higher concentration of positively charged NPs (>5 nM) caused oxidative stress and cell death. Thus, the charge property of NP carriers should be also optimized to improve colloidal stability and interaction with target cells, and, therefore, effective exogenous delivery of antigen molecules.

#### 4.1.2. Polymer-Based Materials

Among the various template materials used to fabricate NP cores, polymer-based NPs have shown a series of technical advantages for carrier development. Such improvements in functional polymeric NPs for TCL delivery include the controllability of the sustained release of various TCL cargos, cargo-protective efficacy through encapsulation, increased half-life and bioavailability of antigens, and a compatibility with vaccine adjuvant delivery, which is beneficial for inducing long-lasting immunity [109].

Poly(lactic-co-glycolic acid) (PLGA) NPs have become a popular candidate for drug delivery systems due to their biodegradability via hydrolysis and easy surface functionalization [110,111], and they can also be used for the delivery of antigens or adjuvant to improve DC-mediated immune responses [112]. Table 2 summarizes the TCL delivery platforms using polymer-based materials.

**Figure 4:**
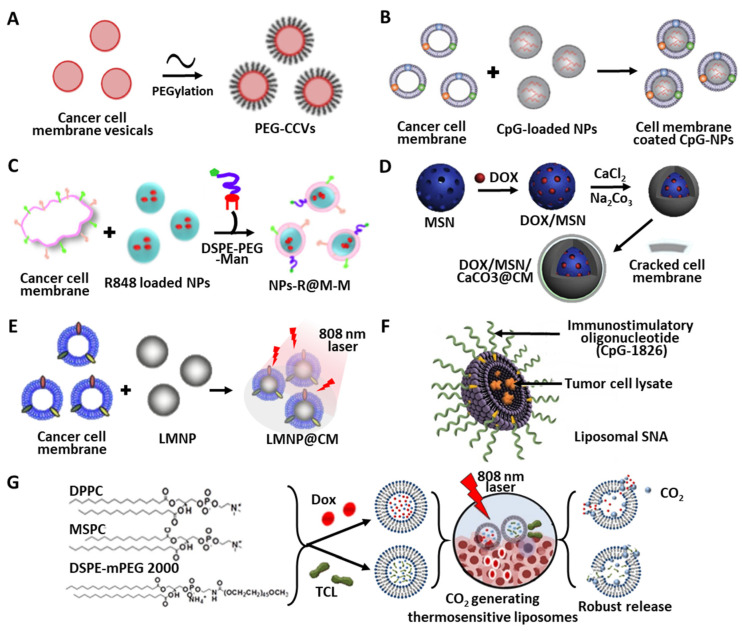
Schematic illustration of various biomaterial-based TCL delivery platforms. (**A**) PEGylated cancer cell membrane vesicles (CCVs) for steric stabilization, (**B**,**C**) PLGA nanoparticle-mediated delivery, (**D**) cancer cell membrane-coated inorganic material-based designs, (**E**) cell membrane-coated liquid metal nanoparticle with NIR irradiation, (**F**,**G**) cargo encapsulated within liposomal nanoparticles with lipid-mediated surface modification (All figures were reproduced with permission from Refs. [26,114,115,116,119,120,121]).

The potential application of PLGA NPs loaded with gastric TCLs for antigastric tumor immunotherapy has been demonstrated [113]. In this instance, TCLs were prepared from primary gastric tumor cells obtained from gastric cancer patients, and encapsulated into PLGA NPs as DC antigen delivery vehicles. Upon delivery to mDCs, a higher expression of HLA-DR and co-stimulatory molecules (e.g., CD80 and CD86), and increased levels of IL-12 and IFN-γ were achieved than from bolus TCL treatment, leading to Th1 immune system pathway activation and augmented T lymphocyte proliferation. In addition to the conventional advantages of polymeric NPs for TCL delivery, PEGylation can also be applied to increase the retention time of therapeutic antigens, thus avoiding in vivo degradation by various proteases, and providing steric stabilization through the formation of a hydration layer on the particle surfaces [117]. Flexible PEG linker-mediated functionalization of NP surfaces also facilitates the adjustment of the chain length to improve cell recognition and uptake [118]. PEGlyated cancer cell membrane vesicles (PEG-CCVs) have also been developed to enhance serum stability and efficient trafficking to lymph nodes (Figure 4A) [26]. This PEGlyation was carried out using 5 kDa DSPE-PEG, and the resulting PEG-CCVs maintained in vitro stability (i.e., size and PDI) in 10% fetal bovine serum (FBS) conditions for 3 days at 37 °C, as well as in vivo draining efficiency to local lymph nodes upon subcutaneous administration.

#### 4.1.3. Camouflage Using Cancer Cell Membranes

As previously discussed, endogenous plasma membranes from whole TCLs are a good source as antigens, mimicking the surface architecture of cancer cells and inducing interplay with immune cells by the presence of membrane-bound tumor antigens. Hence, the artificial coating of cancer cell membrane components onto NP surfaces has also been developed. Such PLGA NPs covered with cancer cell membranes (CCNPs) exhibited colloidal stability and longer circulating properties, and effectively trained the immune system to recognize and fight tumors [122]. In addition, the incorporation of cancer cell membranes onto CpG-containing NPs showed synergistic anticancer vaccination efficacy (Figure 4B) [114]. The concurrent presentation of both immunostimulatory tumor antigens and adjuvant could enhance the effective antigen presentation and the activation of downstream immune processes. Based on the facilitated expression level of co-stimulatory receptors on DCs, cancer cell membrane-associated specific antigen presentation, and higher CD8^+^ T cell proliferation to recognize specific melanoma antigens (i.e., gp100 and TRP2), in vivo vaccination resulted in survival rates of 86% in a B16-F10 tumor model with mice.

Similarly, the combination of a mannose (Man) moiety and a TLR 7 agonist (R837) with CCNPs (Man-R837-CCNPs) showed enhanced cellular uptake and antitumor immune responses (Figure 4C) [115]. Through (1) specific binding between Man and its receptors on DCs, (2) activation of innate immunity by R837 adjuvant, and (3) stimulation by melanoma cell membranes, BMDCs treated with Man-R837-CCNPs achieved higher maturation, with the enhanced expression of CD80 and CD86 and the significantly increased secretion of cytokines (IL-12p40 and TNF-α). Although template CCNPs, R837-loaded PLGA NPs without membrane coating, and R837-CCNPs without a Man moiety slightly inhibited tumor progression compared to untreated controls in B16-OVA tumor models, Man-R837-CCNPs exhibited the strongest antitumor efficacy and vaccination through homotypic targeting mediated by cancer cell surface antigens, and increased numbers of CD8^+^ T cells.

#### 4.1.4. Inorganic Templates for TCL Delivery

In addition to polymeric NPs, inorganic porous particles, such as calcium carbonate (CaCO_3_) and mesoporous silica NPs (MSNs), have also been used as templates for the encapsulation of proteins and peptide antigens. Lybaert et al. [23] utilized CaCO_3_ particles covered with a polymeric TLR7/8 agonist (CL264) to encapsulate TCLs. CaCO_3_ particles with highly porous inner architecture showed a high loading capacity for macromolecules via surface adsorption and encapsulation into the inner core. Surface coating with polycations of the copolymer of N -(hydroxypropyl) methacrylamide (HPMA) and N-(3-aminopropyl) methacrylamide (APMA) modulated the surface charges to adsorb the TLR 7/8 agonist by the combination of electrostatic interaction and physisorption. Additionally, TCLs were prepared from the Lewis lung cancer cell line expressing OVA antigens, coprecipitated with CaCl_2_ and Na_2_CO_3_ during the fabrication of CaCO_3_ particles, and were encapsulated into the core.

The delivered OVA-containing TCLs using TCL-TLR-CaCO_3_ particles resulted in the cross-presentation of OVA by DCs after the migration of the particles into phagosomes and fusion with acidic lysosomes [123]. The results of the co-delivery of tumor-associated antigens using TCLs and the TLR7/8 agonist indicate the higher efficiency of cross-presentation and in vivo antitumor responses via enhanced immunogenicity, compared to any single treatment.

Since TLR is one of the PRRs in DCs [124], this co-delivery strategy using TCLs-TLR-CaCO_3_ particles could (1) activate PRRs by pathogen-associated molecular patterns (PAMPs) and DAMPs derived from necrotic cells (i.e., TCLs), and (2) upregulate antigen presentation by the additional efficacy of the TLR agonist as a potent activator.

A similar surface coating was also used to fabricate cancer cell membrane-coated MSNs [119]. Likewise, the chemotherapeutic drug doxorubicin (DOX) was entrapped in the inner porous structure of the MSNs (i.e., DOX-MSNs), and membrane fragments from LNCaP-AI prostate cancer cell lines (CMs) were then adsorbed onto the DOX-MSNs (i.e., DOX-MSN-CM) (Figure 4D). Along with the induced apoptosis of prostate cancer cells, the co-administration of DOX and CMs using MSNs significantly suppressed tumor growth in LNCaP-AI tumor models.

Recently, liquid metal (LM) has also been utilized as a template core for the development of a nanovaccine for tumor prevention [116]. In this study, CMs derived from 4T1 murine breast tumor cells were coated onto mPEG_5000_-SH-modified eutectic gallium–indium LM NPs (Figure 4E). As well as the antigenic efficacy of CMs and the immune adjuvant effect of LM, the additional photothermal conversion efficacy of LM NPs irradiated by an 808-nm laser facilitated local inflammation, and the subsequent recruitment of APCs, by the increased secretion of pro-inflammatory factors (i.e., IL-6 and TNF-α) and metal-induced NF-kB immune activation pathways [125]. In addition to the effective in vivo delivery of antigens to lymph nodes, three vaccinations within 15 days before the inoculation of 4T1 tumor cells in a mouse model also indicated the significant tumor prophylactic efficacy of CM-coated LM NPs with laser irradiation.

#### 4.1.5. Adjuvant Activities of NPs

Some materials have shown potent adjuvant efficacy to stimulate cellular immunity and modulate immune responses. For instance, aluminum phosphate (AP) was discovered in 1926 as an adjuvant, and was later approved by the United States FDA [126,127]. Therefore, aluminum-containing adjuvants could also be used as cancer vaccines by antigen adsorption via electrostatic attraction and ligand exchange. In particular, CpG-loaded AP NPs coated with B16F10 tumor cell membranes have been developed for cancer vaccination in melanoma models [35]. Again, the surface-incorporated cancer cell membranes enhanced the colloidal dispersion of AP NPs and functioned as native tumor antigens. The dual functions of the AP-mediated adjuvant effects and immunogenicity of antigens effectively mDCs activation, improved lymph node targeting, and facilitated strong tumor-specific cellular immune responses after subcutaneous injection in mice.

Chitosan, a cationic polysaccharide, is also widely used as a vaccine delivery vehicle due to its adjuvant efficacy to promote IFN secretion in mature bone marrow-derived cells (BMDCs), and thus, enhances antigen-specific Th1 responses [128]. Chitosan adjuvants delivered to DCs could induce mitochondrial stress and generate ROS. Subsequent activation of the cGAS-STING pathway triggers the production of type I interferons, and further DC maturation occurs. In addition to the adjuvant effect of chitosan, the Man-based surface functionalization of core chitosan NPs (Man-CTS NPs) facilitates the targeting efficacy of TCL delivery to APCs by binding to Man receptors located on DC membranes [22]. This Man coating also enhances the in vitro bone marrow DC uptake of antigens in TCLs through receptor targeting [129]. Therefore, treatment using B16 melanoma TCL-loaded Man-CTS NPs augmented DC maturation and the related antigen presentation, indicated by the enhanced expression levels of surface markers (i.e., MHC I, MHC II, CCR7, CD80, CD86, and CD40) in vitro and in vivo. An elicited adjuvant effect and T cell priming were further observed with the increased proliferation of both CD8^+^ and CD4^+^ T cells, and the upregulated expression levels of serum IFN-γ and IL-4, confirming in vivo T cell activation in melanoma mice models. Vaccination efficacy and therapeutic effects of TCL-loaded Man-CTS NPs were proven by tumor growth inhibition and reductions in tumor weight.

Additionally, the neurotransmitter dopamine (DA) has also been used for the immune system activation of effector T cells and the suppression of regulatory T (Treg) cells by reacting with DA receptors. DA activates NF-κB to upregulate pro-inflammatory cytokines and chemokines (e.g., IL-6, IL-1β, IL-18, CCL2, and CXCL8) [130]. Wang et al. synthesized polydopamine (PDA)-based NPs covalently conjugated with colorectal cancer TCLs (TCL@PDA NPs) by the interaction between catechols in DA and the amine/thiol groups of antigens in TCLs [24]. PDA-based NPs showed potential as an antigen carrier, exhibiting (1) PDA-mediated pro-inflammation, with increased secretion of IFN-γ and TNF-α, and (2) DC maturation, with the enhanced expression of MHC II and secretion of Th1-related cytokines. In a C57BL/6 mouse model, three (day 4, 10, and 18 after cancer inoculation) subcutaneous vaccinations with TCL@PDA NPs significantly increased the subpopulations of CD4^+^ and CD8^+^ T cells in the spleen and LNs, as well as the memory T cell response. Therefore, both in vivo antitumor efficacy and tumor prevention effects were sufficiently achieved by the combination of PDA and TCLs.

### 4.2. Liposome

Liposomes are another type of exogenous TCL delivery platform. Due to the characteristic structure and composition of liposomes, the entrapment of hydrophilic cargo into the inner core of the liposomes, and additional lipid-mediated surface modification with functional moieties, are possible [131]. Based on these liposomal design strategies, Callmann et al. developed TCL-loaded liposomal spherical nucleic acids (Lys-SNAs) (Figure 4F) [120]. For their fabrication, TCLs from triple-negative breast cancer cells were encapsulated in the core of liposomes, while cholesteryl-modified immunostimulatory oligonucleotide adjuvants (CpG-1826) were immobilized on the surface. As described in the previous section, the oxidation of tumor cells prior to lysate generation using HOCl (OxLys) increases immunogenic aldehyde-modified antigens. After peritumoral administration into an EMT6 mouse mammary carcinoma model, OxLys-SNAs significantly increased the population of cytotoxic CD8^+^ T cells, and simultaneously decreased that of myeloid derived-suppressor cells within the tumor microenvironment compared to Lys-SNAs and simple mixtures of OxLys. The enhanced therapeutic efficacy of the OxLys-SNA formulation was also indicated by antitumor activity, prolonged survival, and the inhibition of tumor regeneration. Therefore, the proper packaging and presentation of adjuvant and human-specific TCL-derived antigens into the liposomal structure is also an important design parameter for exogenous TCL delivery.

In addition to tumor-specific antigen delivery, leading to the maturation and activation of DCs, additional functions of liposomal carriers could facilitate immune modulatory responses. Won et al. [121] developed CO_2_-generating thermosensitive liposomes (BG-TSLs) that encapsulate melanoma-derived whole TCLs (Figure 4G). The lipid layers (a combination of DPPC/MSPC/DSPE-mPEG 2000) of these liposomal TCL carriers were fabricated using a thin lipid film hydration method [132]. Triggering TCL payload release by external near-infrared (NIR) irradiation increased anticancer responses through effective antigen presentation and maturation of DCs, T cell activation, and the proliferation of cytotoxic CD8^+^ T cell populations. Moreover, CO_2_ bubbles generated by the decomposition of the NH_4_HCO_3_ co-payload enhanced the expression of pro-inflammatory cytokines, and suppressed tumor growth in tumor-bearing C57/BL6 mice models. Therefore, the combination of multiple cargo molecules with TCLs and the stimuli-responsive modulation of the liposomal architecture could be employed not only for in vivo DC activation, but also for therapeutic anticancer treatment with CpG-1826, which showed complete tumor remission after 100 days in 45% of the animals tested.

### 4.3. 3D Polymeric Gel

**Figure 5:**
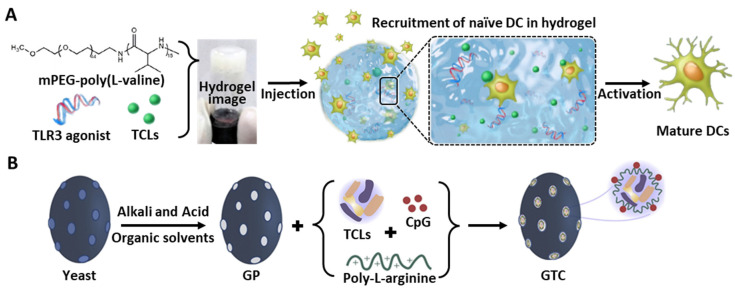
Schematic illustration of TCLs and adjuvant co-delivery using exogenous delivery platforms. (**A**) After injection of TLR3 agonist and TCL-loaded mPEG-poly(L-valine) hydrogels, naïve DCs aggregate around hydrogel. (**B**) Natural component, β-glucan particle (GP)-based TCL and CpG delivery. All figures were reproduced with permission from Refs. [28,133].

The hydrogel-mediated co-delivery of multiple immune modulators has also been investigated. As an injectable vaccination platform, Song et al. developed poly(L-valine) (PEV)-based 3D peptide hydrogels for the co-delivery of melanoma-derived TCLs and a TLR3 agonist (Figure 5A) [133]. The sustained release of both tumor antigens and immune potentiators promoted DC maturation. The injected peptide hydrogel was able to maintain the localization of encapsulated TCLs at the in vivo vaccination site, and the expression of CD86 and MHC II antigens on DCs, and the CD8^+^ T cell response, was significantly elevated compared to the administration of free TCLs or gels without the agonist molecule. Further tumor suppression also suggests that the formulation of peptide hydrogels encapsulated with TCL-derived tumor antigens and a TLR agonist could be utilized as a cancer vaccine platform.

A similar peptide hydrogel formulation has also been applied for the delivery and in vivo localization of multiple immune stimulants. mPEG-poly (L-alanine) (PEA)-based injectable peptide hydrogel could effectively encapsulate (1) melanoma-derived TCLs, (2) GM-CSF, and (3) dual immune checkpoint inhibitors (anti-CTLA-4/PD-1 antibody) during the spontaneous self-assembly of the polypeptide and subsequent gel formation via hydrophobic interactions [25]. Hence, persistent and synergistic DC activation by released TCL antigens and GM-CSF was achieved in C57BL/6J mice models with enhanced T cell responses. Especially, the augmented expansion of effector CD8^+^ T cells within the spleens and tumors of immunized mice by immune checkpoint blockade was observed. This hydrogel-based combination therapy showed superior immune modulation and anticancer efficacy compared to any single cargo delivery, demonstrating prolonged in vivo antigen-specific T cell immune responses.

Furthermore, cryogels (i.e., supermacroporous polymeric network obtained from the ice crystal formations through the steps of phase separation, crosslinking, and polymerization [134]) were also developed as a similar co-delivery platform for the in vivo administration of GM-CSF (DC enhancement factor) and CpG ODN (DC-activating factor) [135]. This cryogel-mediated vaccination platform effectively enhanced DC activation and leukocyte recruitment, and showed higher survival rates in melanoma-challenged C57BL/6 mice models than bolus treatment with immunoactive factors.

### 4.4. Natural Components

Some natural compounds possess sufficient adjuvant efficacy to trigger DC activation. Previous studies have used LPS, a membrane component of Gram-negative bacterial cell walls, because of its adjuvant effect on the activation of TLR4 signaling pathways and the CD4^+^ T cell response [136]. Hence, a series of studies have investigated exogenous signaling via TLR4 on immune cells, and have tried to design TLR4 agonists as vaccine adjuvants [137,138,139]. LPS was reported to interact with TLR-4 in DCs, inducing multiple intracellular signaling cascades to express extracellular signal-regulated kinase, c-Jun N-terminal kinase, p38 mitogen-activated protein kinases, and NF-κB, and affected the production of IL-12 [140]. However, the single-use of LPS for immune activation might evoke vaccine reactogenicity, and induce improper signaling direction for DC activation and further vaccination [141,142].

Despite LPS-mediated immune activation, a high level of immunosuppressive cytokine secretion (such as IL-10) is usually observed. Therefore, other cellular components in bacterial cells could be used for the upregulated expression of immunoactivators, with reductions in immunosuppressive cytokines to deliver the TCLs [27]. For example, the empty envelope of Gram-negative bacteria (i.e., bacterial ghosts (BGs)) with intact cell surface structures exhibited strong adjuvant properties for the induction of DC maturation, and carried TCLs as immune adjuvants in the empty inner core. Facilitated by co-administration with IFN-γ, these TCL-loaded BGs showed superior DC maturation (i.e., upregulated expression of DC maturation markers, including CD86, CD80, and MHC II) compared to treatment with LPS. The secretion of Th1-polarizing cytokine IL-12p70 in DCs was also increased by TCL-loaded BGs with IFN-γ, whereas the level of pro-tolerogenic cytokine IL-10 was decreased. Moreover, the expression of immunoglobulin-like transcript 3, an inhibitory receptor used to establish suppressor T cells by inducing tolerance [143], was also decreased in DCs treated with TCL-loaded BGs. These results demonstrate that the TCL-loaded BGs could potentially overcome immunosuppressive and pro-tolerogenic effects on various cancer types as an effective inducer of Th1-polarized CD4^+^ and associated CD8^+^ T cell-mediated antitumor immunity.

The β-glucan particles (GPs) derived from yeast (e.g., *Saccharomyces cerevisiae*) are another example of natural compound-based fabrication of a TCL carrier (Figure 5B) [144]. Since the 1,3-β-glucan outer shell can provide receptor-mediated phagocytic uptake by cells expressing β-glucan receptors, GPs can be used for the APC-targeted delivery of soluble payloads [145]. Various potential functions of GPs, such as the stimulation of pathogens invading the body, sustained antigen release, facile internalization into APCs, and PAMP-like signaling, could induce robust immune activation. Through a similar encapsulation of antigens into the inner hollow cavity of GPs, the induction of safe immunogenicity by an engineered pathogen-mimicking system, and long-term interaction via the sustained release of cargo antigens, could be achieved. Therefore, Hou et al. [28], developed GPs encapsulating murine colon adenocarcinoma cell (MC38) lysates with additional stimulation provided by a CpG TLR9 agonist. In addition, the co-incorporation of poly-L-arginine improved the protection against challenge from live tumor cells in animal models when co-injected with tumor antigens, and also promoted the in vivo charging of MHC II^+^ APCs [146,147]. This GP platform was internalized in up to 70% of the DCs by energy-dependent and dectin-1 receptor-mediated endocytosis, and the sustained release of the cargos resulted in the significantly higher expression of CD86 than that of the LPS controls. Moreover, NLR pyrin domain-containing protein 3 inflammasome-mediated DC activation was also confirmed by increased cleaved caspase-1 p10 (10 kDa) levels in GP-treated BMDCs, and the correlated IL-1ß secretion [148]. A summary of whole-TCL delivery platforms using liposomes, 3D polymeric gel, and natural components are indicated in Table 3.

### 4.5. Future Progress of Cancer Immunotherapy Using TCLs

The study of the relationship between cancer and immune responses has increased rapidly over the last few decades, among which TCLs have demonstrated their utility to elicit sustained CTL responses and vaccine effectiveness in cancer therapy. Moreover, since TCLs do not induce a strong enough CTL response against cancer, additional immune agonists or adjuvants have been utilized in combination, as previously described [149]. A series of delivery platforms described in this review possess the necessary functionalities, including an effective cargo protective carrier, immune agonistic property, and/or adjuvant efficacy. However, it should be also considered that there might be a possible risk of overreaction, such as cytokine storm activation, during periods of high immune activity [150]. Therefore, in order to develop more effective strategies in TCL delivery, the optimization for clinical safety, and the combination with an additional immune agonist or adjuvant, is necessary for inducing selective activation of T cells to respond to specific tumor antigens, rather than broad activation of various immune cells, which could cause deleterious side effects [151]. It should be also emphasized that there is still work to be done in developing combination therapy and optimizing vaccine platforms before TCL-based treatment becomes a viable immune modulatory and therapeutic strategy [152].

## 5. Conclusions

TCL-mediated cancer immunotherapy has been shown to involve the activation of tumor-specific CD8^+^ and CD4^+^ T cells via a vast array of immunogenic epitopes. However, an in-depth understanding of the physiological functions of DCs and in vivo interactions with other immune cell populations are needed to improve therapeutic effectiveness and establish optimal modulation in adaptive immunity. To emphasize the efficacy of TCL-mediated anticancer therapy, we reviewed (1) various experimental methods for preparing TCLs as a major immunomodulatory source, (2) TCL-mediated augmentation in DC-T cell interaction, and the subsequently induced activation of T cells, and (3) the recent progress in the biomaterial-based in vivo administration of TCLs. With the aid of co-stimulatory adjuvants, biomaterial-mediated exogenous TCL delivery could be an efficient therapeutic strategy to enhance the stability and sustained release of cargo TCLs, improve the specificity of DC targeting, and activate DCs synergistically. As a result of sufficient DC activation (i.e., increased antigen presentation and cytokine release), antigen-specific T cell-mediated tumor suppression and vaccination can be upregulated through the dynamic interplay of immune responses. Therefore, exogenous TCL delivery techniques could be a promising treatment for enhancing the DC-mediated activation of adaptive immune responses, vaccination, and tumor-specific suppression.

## Figures and Tables

**Table 1:** Condition for preparation of TCLs.

Classification of Process		Condition	Ref.
Physical disruption	Freeze–thaw cycle	Freeze at −80 °C and thaw at 37 °C (repeat)	[22,23,24,25,26,27,28]
	Sonication	Sonicate 3 times for 10 s	[29]
	UV irradiation	Irradiate with 1500 μW/cm^2^ UVB	[30]
Pretreatment of source tumor cells	Heat shock	1. Treat at 42 °C for 1 h and 37 °C for 2 h 2. Additional physical disruption	[31,32]
	CD47 agonist	Treat 150 or 300 μM of PKHB1 for 2 h	[33]
	*Phyllanthus amarus*	1. Treat 1000 μg/mL *Phyllanthus amarus* 2. Additional physical disruption	[34]
Cell membrane isolation	Sucrose-dependent	1. Mix 0.0759 M sucrose and 0.225 M D-mannitol-containing buffer 2. Centrifuge at 10,000× *g* for 25 min 3. Centrifuge the supernatant at 150,000× *g* for 35 min	[10]
	Sucrose-independent	1. Centrifuge at 10,000× *g* for 25 min 2. Centrifuge the supernatant at 150,000× *g* for 40 min	[35]

**Table 2:** Polymer-based material delivery platforms for exogenous TCL delivery.

Material	TCL Type	Specificity	Material Platform	Target Cancer	Outcome	Ref.
PLGA	Whole TCLs	Human	TCL-loaded PLGA NPs	Gastric cancer	Increased IL-12 and IFN-γ in DCs Th1 immune system pathway activation	[113]
	CM	Mouse	Cell membrane coated-CpG-PLGA NPs	Melanoma	Stability and longer circulation High recognition of specific tumor antigens 86% survival in vaccination group	[114]
	CM	Mouse	Cell membrane coated-R848-PLGA NP–mannose moiety conjugate	Melanoma	Specific binding by mannose Homotypic targeting on cancer cell surface antigens	[115]
PEG	CM	Mouse	Co-delivery of PEGylated cell membrane and CpG	Melanoma	Enhanced serum stability Efficient trafficking to LNs 63% tumor regression	[26]
PEGylated LM	CM	Mouse	Cell membrane coated-PEG-LM NPs	Breast	Immune adjuvant effect and photothermal conversion efficacy with irradiation Metal-induced NF-kB immune pathway activation	[116]
CTS	Whole TCLs	Mouse	Mannose-coated TCLs-CTS NPs	Melanoma	Mitochondrial stress, ROS generation, and cGAS-STING pathway activation Improvement in NP uptake efficacy	[22]
PDA	Whole TCLs	Mouse	TCL-loaded PDA NPs	Colorectal cancer	Reacted with dopamine receptor Increased the subpopulation of T cells	[24]

PLGA, poly(lactic-co-glycolic acid); TCL, tumor cell lysate; IL, interleukin; IFN, interferon; Th, T helper cell; CM, cell membrane; PEG, polyethylene glycol; R848, resiquimod; LN, lymph node; LM, liquid metal; CTS, chitosan; ROS, reactive oxygen species; PDA, polydopamine.

**Table 3:** Biomaterial-mediated whole-TCL delivery platform.

Platform	Material	Specificity	Material Platform	Target Cancer	Outcome	Ref.
Liposome	Liposomal spherical nucleic acids	Mouse	CpG-1826-coated and TCL-loaded liposome	Triple-negative breast cancer cell	Increased population of CTLs Decreased population of myeloid derived suppressor cells	[120]
	CO_2_-generating thermosensitive liposomes	Mouse	Co-delivery of DOX-loaded liposome and TCL-loaded liposome	Melanoma	High expression of pro-inflammatory cytokines and suppressed tumor growth by external NIR irradiation and generated CO_2_ bubbles	[121]
3D polymeric gel	PEV-based hydrogel	Mouse	TCL- and TLR3-loaded PEV hydrogel	Melanoma	Localization of injectable hydrogel and induction of sustained release Highest percentage of CTLs in LN	[133]
	PEA-based hydrogel	Mouse	TCL, GM-CSF, and anti-CTLA4/PD-1 Ab-loaded PEA hydrogel	Melanoma	Persistent and synergistic DCs activation Augmented expansion of effector CD8^+^ T cells	[25]
	Cryogel	Mouse	CpG ODN, GM-CSF, and RGD-loaded cryogel-containing TCLs	Melanoma	Enhanced DC activation Leukocyte recruitment Greater survival rates	[135]
Natural component	Empty envelope of bacterial ghost	Human	Combination of TCL-loaded bacterial ghost and IFN-γ	Melanoma, renal cell carcinoma, glioblastoma	Decreased expression of ILT3 and inhibitory receptor	[27]
	Yeast derived ß-glucan particle	Mouse	TCL, CpG, and poly-L-arginine-loaded ß-glucan	Colorectal cancer	High internalization in DC NLRP3 inflammasome-mediated DC activation	[28]

3D, three dimensional; CTL, cytotoxic T lymphocytes; DOX, doxorubicin; TCL, tumor cell lysate; NIR, near-infrared radiation; TLR, Toll-like receptor; PEV, poly(L-valine); PEA, poly(L-alanine); LN, lymph node; GM-CSF, granulocyte-macrophage colony-stimulating factor; Ab, antibody; DCs, dendritic cells; RGD, Arg-Gly-Asp; IFN, interferon; ILT3, immunoglobulin-like transcript 3; NLRP3, nucleotide-binding oligomerization domain 3.

## Data Availability

The data presented in this study are contained within the article.

## Abbreviation

Th	T helper
APC	Antigen-presenting cell
CTL	Cytotoxic T lymphocyte
IFN	Interferon
IL	Interleukin
DC	Dendritic cell
MHC	Major histocompatibility complex
TCR	T cell receptor
iDC	Immature DC
mDC	Mature DC
TNF	Tumor necrosis factor
TAA	Tumor-associated antigens
TCL	Tumor cell lysate
DAMP	Damage-associated molecular patterns
PRR	Pattern recognition receptor
HSP	Heat shock protein
HMGB-1	High-mobility group box-1
TLR	Toll-like receptor
UV	Ultraviolet
ICD	Immunogenic cell death
HOCl	Hypochlorous acid
OVA	Ovalbumin
SqA	Squaric acid
TSP-1	Thrombospondin-1
ROS	Reactive oxygen species
LPS	Lipopolysaccharide
ER	Endoplasmic reticulum
TAP	Transporter associated with antigen processing
FDA	Food and Drug Administration
GM-CSF	Granulocyte-macrophage colony-stimulating factor
MAGE-1	Melanoma-associated antigen-1
NP	Nanoparticle
VEP	Virus envelope protein
PLGA	Poly(lactic-co-glycolic acid)
PEG-CCV	PEGlyated cancer cell membrane vesicle
FBS	Fatal bovine serum
CCNP	Cancer cell membrane nanoparticle
Man	Mannose
CaCO_3_	Calcium carbonate
MSN	Mesoporous silica NP
HPMA	N -(hydroxypropyl) methacrylamide
APMA	N-(3-aminopropyl) methacrylamide
PAMP	Pathogen associated molecular pattern
DOX	Doxorubicin
LM	Liquid metal
AP	Aluminum phosphate
BMDC	Bone marrow dendritic cell
DA	Dopamine
PDA	Polydopamine
Lys-SNA	TCL-loaded liposomal spherical nucleic acid
CpG-1826	Cholesteryl-modified immunostimulatory oligonucleotide adjuvants
BG-TSLs	CO_2_-generating thermosensitive liposomes
NIR	Near-infrared
PEV	Poly(L-valine)
PEA	Poly(L-alanine)
BGs	Bacterial ghosts
GPs	ß-glucan particles
MC38	Murine colon adenocarcinoma cell
